# Strength Characteristics of Historical Mortars—Experimental Study Using the Double Punch Method

**DOI:** 10.3390/ma18214868

**Published:** 2025-10-24

**Authors:** Piotr Matysek, Michał Witkowski

**Affiliations:** Faculty of Civil Engineering, Cracow University of Technology, 31-155 Krakow, Poland; witkowskipk@tlen.pl

**Keywords:** brick masonry, historical mortars, strength characteristics, double punch test

## Abstract

Identification of the strength characteristics of mortars in brick or stone masonry is crucial in the structural analysis of heritage buildings and selecting materials for their repairs and reconstruction. Non-destructive, minimally destructive, and minor-destructive tests have been developed to establish the strength of mortar in existing masonry. This paper presents strength tests on mortar samples extracted from bed joints of heritage buildings erected in the historic center of Cracow during the 19th and 20th centuries. The mortar samples were tested using the double-punch method, a minor-destructive technique especially useful for heritage structures where cutting out large masonry specimens is not possible due to conservation reasons. The impact of sample thickness and type of capping materials on the test results were analyzed in detail. Practical recommendations are also proposed for the procedure of the double-punch method in relation to historical mortars.

## 1. Introduction

Bricks, stone blocks, and mortars were basic materials used in the construction of heritage masonry buildings. Over the centuries, very different types of bricks, stone blocks, and mortars, as well as various techniques for making masonry, were employed. For this reason, the mechanical characteristics of masonry varied substantially. The assessment of these characteristics is one of the primary tasks in the process of structural analysis of heritage buildings. The basic characteristic to be assessed is the masonry compressive strength, which depends primarily on the strength of the bricks, stone blocks, and mortars, as well as on the quality of the workmanship of masonry. It follows that mortar strength is one of the important parameters that should be tested and properly assessed based on the results of experiments. Knowledge of the strength of mortars is also of fundamental importance in the process of selecting materials for the repair of heritage buildings that have been damaged [1,2,3,4,5,6].

Identifying the mortar strength in structures is not easy. Only small, irregular samples are usually available from mortar joints. The height of the samples taken from masonry is limited by the thickness of the joints. Therefore, destructive tests on prismatic samples (40 × 40 × 160 mm) recommended in the EN 1015-11 code [7] to establish mortar strength cannot be performed. For this reason, other types of mortar tests are being developed. In historic buildings, non-destructive, minimally destructive, or minor-destructive methods are preferred and used.

Tests are conducted in-situ using Schmidt hammers [8,9,10] or penetrometers dedicated to testing mortars in masonry joints [11,12,13]. Screw (helix) pull-out tests are also performed in the investigation of mortar strength [14,15]. All these experimental techniques are used on the external surface of the masonry. Consequently, they provide information on the mortar strength in the near-surface zones.

More reliable data on the strength characteristics of mortar can be obtained by testing masonry cylindrical samples cut from the structure. In a laboratory, cores are subjected to splitting tests [16,17,18] or are delaminated to prepare mortar samples for destructive tests on small mortar cubes [19,20] or small mortar slabs [17,21,22]. Mortar fragments can also be taken from masonry during planned renovation work—for example, when cutting new openings in the structure. Irregular mortar fragments obtained from masonry walls or pillars are then formatted in the laboratory into small mortar cubes or small mortar slabs.

Testing on small samples cut from mortar joints is currently the most common method. In tests on small mortar cubes, it is necessary to cut out samples with a definite cross section (e.g., 15 mm × 15 mm or 20 mm × 20 mm), which may be difficult and harmful, especially in the case of weak historical mortars. It is much easier to prepare small mortar slabs. The thickness of these slab samples is equal to the thickness of bed joints in masonry, while the horizontal dimensions after regularization are between 40 mm and 60 mm (usually 50 mm). The mortar slabs are loaded using two steel punches with diameters of 20 mm or 25 mm. The stressed area of the samples is located centrally, far from the edges that were subject to the regularization process. Tests on small mortar slabs were developed by J. Henzel et al. [23] and A. Pauser et al. [21]. This method, known as the double punch test (DPT), is described in the German code DIN 18555-9 [24] and UIC 778-3R recommendation [25].

The loading of the mortar samples and failure mode in the DPT method differs significantly from one used in tests of mortar samples according to the EN 1015-11 code [7]. Numerical simulations presented in [17,18] showed that in mortar samples tested using the DPT method, large transverse compressive stresses are created. For this reason, mortar strength tested using the DPT method is usually greater than the mortar strength in uniaxial compression tests.

The interpretation of test results carried out by the DPT is not straightforward. Many factors influence the test results. First and foremost, the geometry of the mortar samples, the diameter of steel punches used in tests, and the type of capping on the samples have a significant influence [26,27,28,29,30,31,32]. Strength tests using the DPT method were conducted primarily on mortar samples extracted from brick masonry made in the laboratory from contemporary materials. The results of these tests indicate the applicability of the DPT method but still require verification in relation to mortars in heritage buildings. Such verification is necessary because historical mortars have slightly different properties than mortars produced today. Different binders, aggregates, and additives were used in the past. To improve mortar strength, many organic and inorganic additives were introduced into mortar compositions. Mortars were mixed, for example, with animal glue, casein, fatty acids, and ground volcanic tuff. A feature of many historical mortars is the presence of large aggregate grains with diameters up to several millimeters. Historical mortars are also characterized by significant water absorption and porosity [4,6,33,34,35].

In this paper, DPT strength tests on mortar samples extracted from bed joints of heritage masonry structures erected in the 19th and first half of the 20th centuries are presented. The research concerns mortars in four structures: a railway bridge, military buildings, and industrial buildings.

The influence of two parameters on the results of mortar tests using the DPT method was investigated. One of these parameters was the thickness of the mortar samples, which resulted from the varying thickness of the joints in the heritage masonry. Based on the analysis of test results, a new formula was developed that is dedicated to historical mortars. This formula allows for taking into account the influence of the thickness of the samples used in the tests on the mortar compressive strength.

The second tested parameter was the method of sample preparation. Two capping materials were used to smooth the sample surfaces: gypsum and quick-setting cement mortar. It was shown that the destructive forces exerted by mortar samples capped with quick-setting cement mortar were, on average, 10% higher than those of samples capped with gypsum.

The paper also presents the results of research on the relationship between the compressive strength of historical mortars and their bulk density. The result of this study is a linear formula that can be used to predict mortar compressive strength based on the bulk density of mortar collected from masonry joints.

## 2. Experimental Research

**Figure 1:**
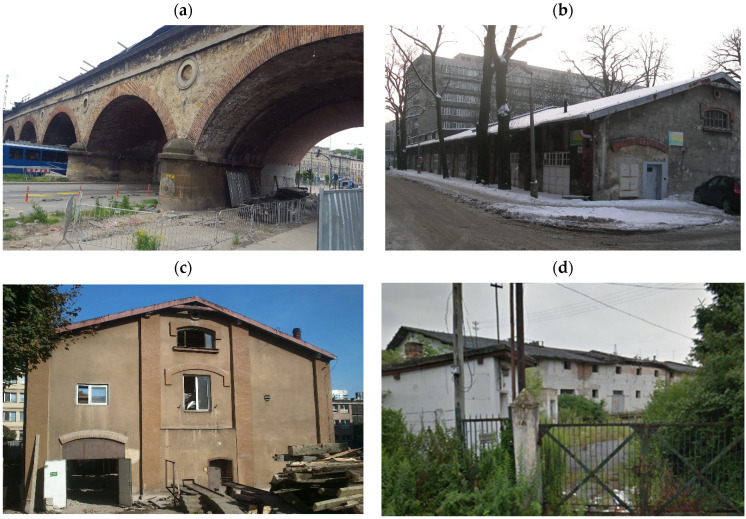
The view of heritage brick structures: (**a**) railway bridge—B1, (**b**) military building—warehouse for ammunition—B2, (**c**) military building—warehouse for cannons—B3, (**d**) industrial building—B4.

The mortar samples used in research were collected from brick masonry structures located within the historic center of Krakow (Figure 1). The arch bridge (see Figure 1a) was built in the 1860s during the construction of the Krakow–Lviv railway connection. The facades shown in Figure 1b,c were storage buildings in the complex of Archduke Rudolf’s Caserns. These military buildings formed part of the Krakow Fortress, which was expanded from the mid-19th century until World War I. The fourth structure (Figure 1d) is part of an industrial complex of grain mills built in the 1930s.

Investigation of mortars taken from heritage facilities was part of the projects aimed at identifying the construction materials used in historic masonry structures. In the bridge, mortar samples were collected from the brick arches, while in buildings B2, B3, and B4, they were collected from the masonry walls. During the examination of buildings B2 and B3, structural fragments were identified that had been erected during the reconstruction works that took place in the 20th century. Therefore, two types of mortar were tested from buildings B2 and B3 (Table 1).

**Figure 2:**
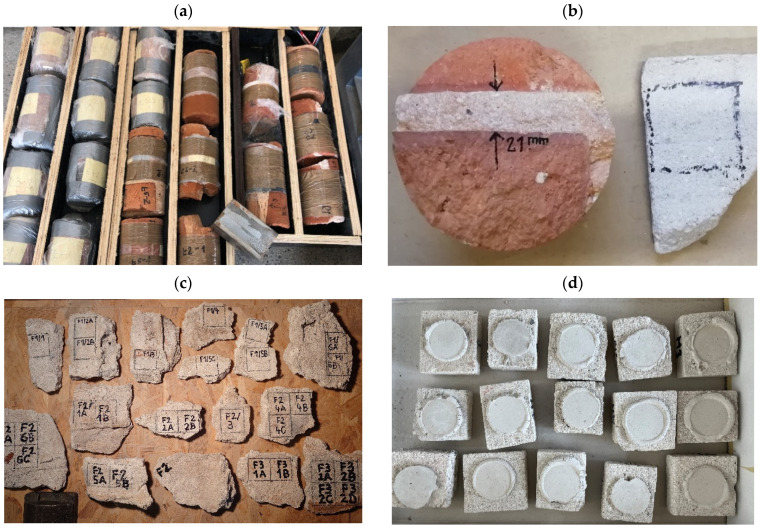
Preparation of mortar samples for DPTs (**a**) masonry cores cut from bridge arches, (**b**) masonry core and irregular mortar slab extracted from central located bed joint, (**c**) irregular mortar slabs extracted from the demolition fragments of brick walls, (**d**) mortar samples after regularization process and after capping.

The mortar samples were collected by means of core drilling through the centrally positioned bed joint (bridge B1) or by splitting the mortar from fragments of brick masonry intended for demolition (buildings B2, B3, B4)—Figure 2.

The joints were split from bricks using a steel chisel. The obtained irregular fragments of the bed joints were used to cut out square specimens with a side length approximating 50 mm. All samples were capped with gypsum or quick-setting cement mortar. The capping process was performed using 30 mm steel punches. After laying the fresh capping material, each sample was pressed into the testing machine with an initial force of about 50 N.

After preparation, all mortar samples were precisely measured. Sample thickness measurement results are given in Table 2. The thickness of each sample is a mean value of measurements at four points around the circumference to an accuracy of 0.1 mm. Finally, 117 mortar samples extracted from historical structures were prepared and measured. Before conducting strength tests, mortar samples were stored for 14 days at a temperature of 20 °C ± 2 °C and humidity of 65% ± 3%.

**Figure 3:**
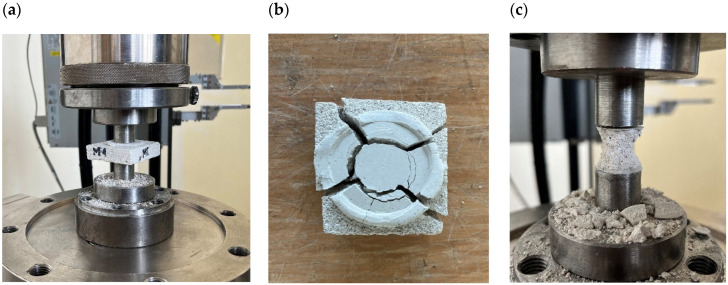
DPT setup (**a**) and typical failure mode of mortar sample (**b**,**c**).

The DPTs were performed according to DIN 18555-9 code [24]. Steel punches with diameter *ϕ_p_* = 20 mm were used. Figure 3 presents an experimental setup and typical failure mode of mortar sample after DPT.

## 3. Test Results

The results of strength tests are presented in Table 3. The strength of mortar obtained in DPTs (*f_j_*) was determined as the ratio of the maximum compression load (*P_max_)* to the steel punch cross-section area (*A_p_*):

(1) $$f_{j}=\frac{P_{max}}{A_{p}}$$

The strengths from tests on mortar samples capped with gypsum were designated as *f_jg_*, while those from tests on samples capped with quick-setting cement mortar were designated as *f_jc_*. Table 3 also provides the maximum aggregate grain size (*d_max_*) identified in samples and bulk density of mortars.

The failure process of the mortar samples, the effects of which are shown in Figure 3, was similar. At high levels of compressive loads, radial cracks appeared in the samples (Figure 3b). As the compressive loads increased, radial cracks enlarged, leading to the detachment of sample parts located outside the directly loaded areas. The final shape recorded after the tests were the typical classic double cones (Figure 3c).

## 4. Discussion

The presented research results concern mortars collected from various types of heritage brick structures. The oldest of these was constructed over 150 years ago. The tested historical mortars were varied in terms of strength properties and bulk density. The compressive strength of the mortars, as determined by the DPT test (*f_j_*), ranged from 2.4 MPa to 18.3 MPa. In the original parts of heritage structures, the strength of the mortars did not exceed 6.0 MPa (mortars m2, m4, m6), while significantly higher strength was achieved by mortars used during the extension or reconstruction of masonry buildings (mortars m3, m5). The compressive strength of the mortar taken from the arch bridge structure was 10.7 MPa, which was close to the strength of mortars in bridges of similar construction [36].

The dispersion of strength test results is large for all types of mortars. The coefficient of variation (CoV) ranged from 0.21 to 0.32. The large dispersion of mortar strength was also observed by A. Drougkas et al. [5] in research conducted on mortar samples extracted from heritage brick pillars and by A. Pauser et al. [21] in tests on brick arch bridges. The high heterogeneity of the mortar in the bed joints of historical structures is the main reason for this phenomenon. The fact that the mortar samples had varying thickness also played a role in the study results. The thickness of the mortar samples depended on the thickness of the joints from which the samples were cut, and these vary greatly. The results given in Table 2 indicate that the thicknesses of the bed joints in heritage structures were also much greater than the requirements of Eurocode 6 [37]. Eurocode 6, which applies to masonry structures, recommends the thickness of bed joints in masonry from 6 mm to 15 mm. The mean thickness values of the tested mortar samples ranged from 16.7 mm to 22.5 mm.

**Figure 4:**
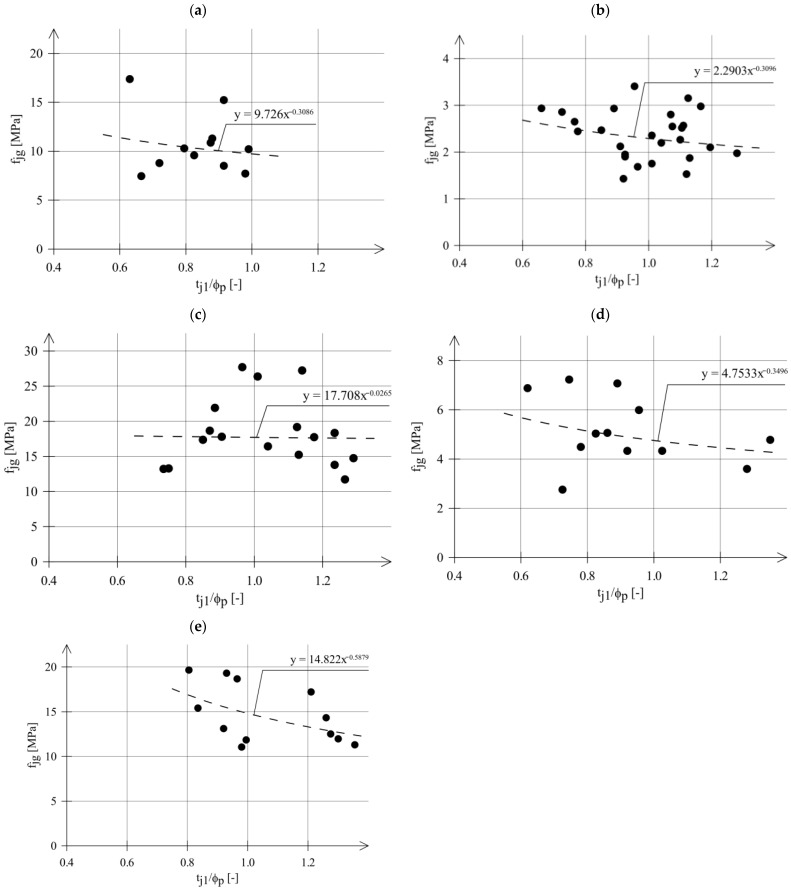
Mortar compressive strength (*f_jg_*) vs. ratio *t_j_*_1_/*ϕ_p_*; (**a**) m1/g, (**b**) m2/g, (**c**) m3/g, (**d**) m4/g, (**e**) m5/g.

The relationships between the strength obtained in the DPT test (*f_j_*) and the ratio of *t_j_*_1_/*ϕ_p_* are presented in Figure 4 for series that included at least 12 tests. Regression functions in Figure 4 are given in the form of power functions:

(2) $$f_{jg}=A\;{\left(\frac{t_{j1}}{\phi_{p}}\right)}^{m}$$

where *t_j_*_1_—thickness of mortar sample; *ϕ_p_*—diameter of steel punches; *A*, *m*—power function coefficients.

The power functions are traditionally used to describe the effect of sample thickness on the strength test results of modern mortars. Power functions were used by E. Sassoni [30] and A. Drdacky [28,29,31]. As shown in Figure 4, the influence of mortar thickness on mortar strength is visible. Generally, lower *f_jg_* values were obtained for larger ratios (*t_j_*_1_/*ϕ_p_*).

This effect depends on the type of mortar—see Table 4.

Only for the highest-strength mortar (m3) did the effect of sample thickness on the strength test results show up to be small, not exceeding a few percent. For the remaining mortars, this effect was significantly greater (from 14% to 32%) and not negligible.

The varied influence of mortar sample thickness on test results for different types of contemporary mortars was observed in tests performed by A. Drdacky et al. [28,29,31] and E. Sassoni et al. [30]. Studies by A. Drdacky et al. and E. Sassoni et al. were conducted on contemporary mortar samples prepared in the laboratory. The dispersions of the results of these tests were clearly smaller than the results of the tests on the historical mortars—presented in Figure 4.

There are several factors contributing to the large dispersion of test results on historical mortars. One of these factors is the influence of the external environment on mortar strength parameters. Mortar samples were collected from various parts of heritage structures, which were subjected to varying degrees of environmental impact and degradation processes. For this reason, the test results may differ significantly.

Another factor that may contribute to the large dispersion of test results is the presence of large-diameter aggregate grains in the relatively small mortar samples. The grains with diameters ranging from 7 mm to 16 mm locally strong changes the properties of the material. This effect is not observed in tests of modern mortars, which do not use aggregates with a grain size larger than 4 mm.

The phenomenon of increasing the value of ultimate forces destroying samples in the DPT test with decreasing sample thickness can be explained on the basis of observations of the form of sample destruction and FE analyses. The typical failure mode of mortar samples are shown in Figure 3b,c. In the DPT method, mortar samples are subjected to a complex stress state generated by local compression, the effects of friction between steel punches and capping material, as well as confinement induced by the unloaded area of the mortar sample.

FE analyses [17,26] showed that horizontal stresses in mortar samples locally compressed by steel punches range from a dozen to several percent of the vertical stresses. The largest transverse compressive stresses in radial and circumferential directions are located directly under loading transferred from capping material to mortar. In the deeper layers of mortar, the confining pressure is less. However, in the free of load sample area the transverse tensile stresses occur, resulting in radial cracks. The failure of the sample is associated also with loss of confinement due to this radial cracking (Figure 3b). The effect of the complex stress state in the mortar sample compressed in the DPT is a typical classic double cone created in the center of the mortar samples (Figure 3c).

In the analytical model presented by A. Benedetti et al. [36], the transverse stresses in the mortar sample were dependent on the value of the friction coefficient between the material of the capping and the steel of the punch through which the load in the DPT test is applied. The greater the friction, the greater the value of the transverse stresses in the mortar sample and the vertical collapse pressures. According to analyses presented in [36], higher destructive compressive forces are expected in tests of samples capped with cement mortar than in tests of samples capped with gypsum.

**Figure 5:**
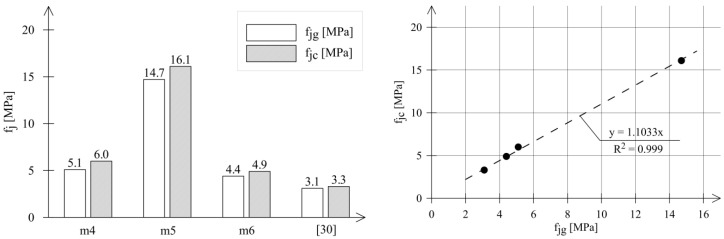
The influence of the capping material on the mortar strength values in the DPTs.

This is confirmed by the results presented in Table 3 and in Figure 5. The strengths determined on gypsum-capped samples were, in each case, lower than those on samples capped with quick-setting cement mortar. The results of the comparative analysis are summarized in Figure 5.

The values of the *f_jc_*/*f_jg_* ratio range from 1.10 to 1.18. Figure 5 also takes into account the research results presented in [30]. The linear regression function has the form:

(3) $$f_{jc}=1103\;f_{jg}$$

The mortar strength determined on samples capped with quick-setting cement mortar is, on average, 10% higher than that of samples capped with gypsum.

From a practical point of view, it is important to convert the results of DPTs into mortar strength in uniaxial compression. J. Henzel et al. [23] tested various types of cement mortars prepared in the laboratory. In each case, the strength determined by the DPT method (*f_jg_*) on 10 mm thick samples was greater than the mortar strength tested on 40 mm prisms (*f_m_*) in uniaxial compression. The *f_jg_*/*f_m_* ratio ranged from 1.1 to 2.2, with a mean value of 1.4. Test results for mortar samples of various thicknesses made from modern materials are given in [26,30]. In [13] the regression function was proposed:

(4) $$f_{m}=\frac{f_{jg}}{1.24{\left(\frac{t_{j2}}{\phi_{p}}\right)}^{-0.69}}$$

From Equation (4), it follows that *f_jg_* equals *f_m_* when *t_j_*_2_ = 27 mm.

**Figure 6:**
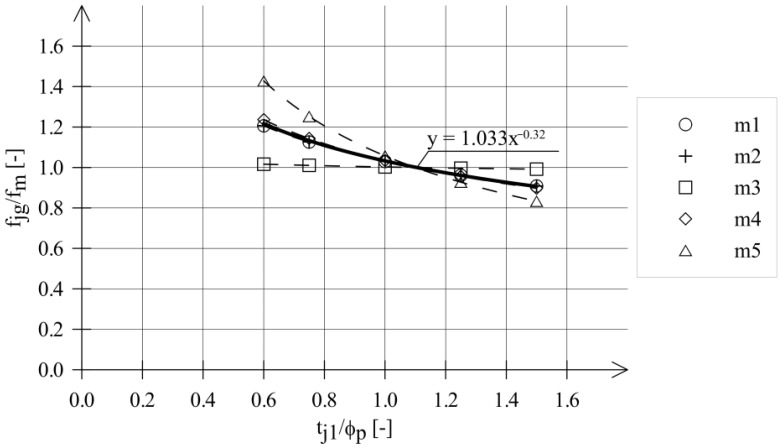
Relationship *f_jg_*/*f_m_* vs. *t_j_*_1_/*ϕ_p_* for historical mortars.

Figure 6 compares the regression functions obtained from tests on historical mortars—see Table 4. It was assumed that the value of *f_m_* corresponds to the compressive strength determined on mortar samples with the thickness of *t_j_*_2_ = 27 mm.

The function derived from analysis of the test results of historical mortar is given below:

(5) $$\frac{f_{jg}}{f_{m}}=1.033\;{\left(\frac{t_{j1}}{\phi_{p}}\right)}^{-0.32}$$

Formula (5) can also be written as follows:

(6) $$f_{m}=\frac{f_{jg}}{1.033{\left(\frac{t_{j1}}{\phi_{p}}\right)}^{-0.32}}$$

The UIC 778-3R recommendations [25] state that, for samples cut from bed joints with the thickness of 10 mm to 25 mm, the following can be assumed:

(7) $$f_{m}=0.7\;f_{jg}$$

Table 5 summarizes the mortar compressive strengths (*f_m_*) calculated on the basis of the DPT test results, Equations (4), (6) and (7).

The lowest *f_m_* values are obtained using Formula (7), which does not take into account the thickness of the mortar samples in the DPT test. Including parameter *t_j_*_1_ in the analysis of strength test results allows for a more precise determination of the strength of historical mortars. The differences between *f_m_* values determined using Formula (4), developed based on the results of tests on contemporary mortars, and Formula (6), based on the presented results of tests on historical mortars, do not exceed 10%.

**Figure 7:**
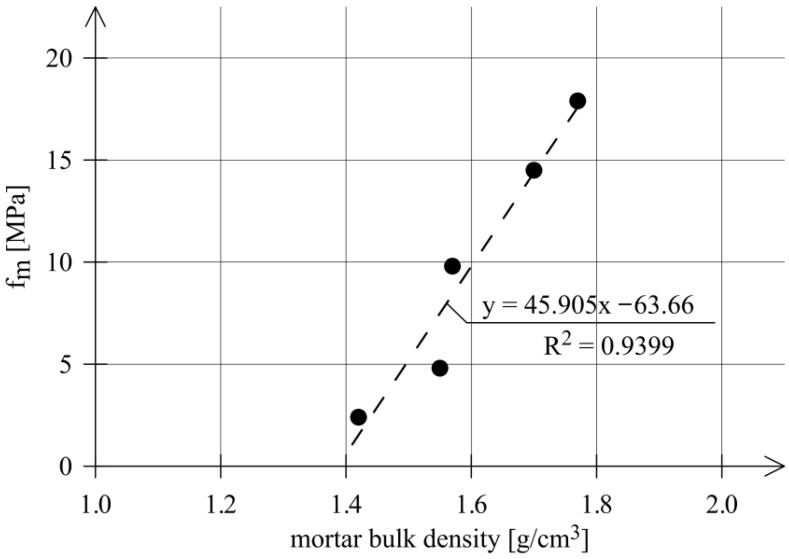
Relationship between the compressive strength of historical mortars *(f_m_*) and their bulk density.

The relationship between the compressive strength of mortars (*f_m_*) and their bulk density is shown in Figure 7. The bulk densities of the tested historical mortars ranged from 1.42 g/cm^3^ to 1.77 g/cm^3^.

As can be seen from the graph in Figure 7, the higher the bulk density of the mortar, the higher its compressive strength. The coefficient of determination (R^2^) for the linear relationship between mortar compressive strength and bulk density is equal to 0.9399.

**Figure 8:**
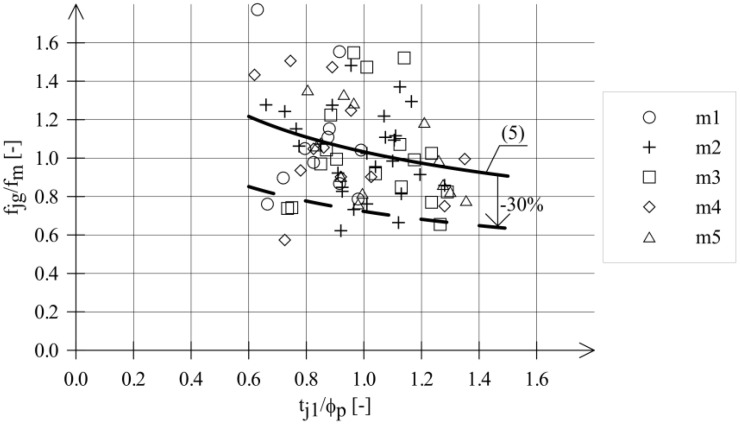
Relationship *f_jg_*/*f_m_* vs. *t_j_*_1_/*ϕ_p_*—test results and formula (5).

The values of *f_m_* given in Table 5 refer to mean compressive strengths of mortars. The large dispersion of test results for historical mortars indicates that mortar strengths, even within the same structure, can vary significantly. The compressive strength value of mortars used in structural analysis of the heritage building should take these differences into account. A proposed solution to this problem could be to use in strength analyses of masonry a corrected value of mortar compressive strength instead of the mean strength. Considering the results presented in Figure 8, a correction factor of 0.7 is proposed for a safe estimation of mortar strength.

## 5. Conclusions

This paper presents the results of tests on mortars collected from heritage buildings located in the historic center of Krakow. The mortar samples were taken from the brick structures of arched railway bridge, military facilities, and industrial building.

The characteristic feature of these brick structures was thick bed joints. The thickness of the bed joints was 20 mm ± 8 mm, significantly greater than the joints in contemporary masonry. In all tested mortars, the presence of large-sized aggregate grains was confirmed. The maximum aggregate grain sizes recorded in mortars ranged from 7 mm to 16 mm. This is a specific feature of mortars used in many heritage buildings in Poland.

The DPT method was used and adopted to determine the strength of historical mortars. The tests were performed on small samples taken from bed joints of masonry. The thickness of the samples was approximately equal to the thickness of the bed joints from which they were cut. The compressive strengths of the mortars obtained in DPTs ranged from 2.4 MPa to 18.3 MPa. The strength tests of all mortars were characterized by the large dispersion of results—the coefficients of variation of the mortar strengths ranged from 0.22 to 0.32.

Due to the significant variation in bed joint thicknesses in the brick masonry, the influence of sample thickness on mortar strength was tested. For all types of mortars, the effect of decreasing mortar strength in DPTs with an increase in sample thickness was visible. Only for one type of mortar did the effect of sample thickness on strength appear to be negligibly small (2%). For others, the effect of sample thickness on the strength test results was significant (from 14% to 32%). Based on the conducted study, the new formula for historical mortar was proposed. This formula enables the analysis of strength test results taking into account the sample thickness parameter.

Also analyzed in the paper was the influence of the capping material on the strength test results. Two types of materials were used for capping: gypsum or quick-setting cement mortar. It was demonstrated that the compressive strengths obtained from testing samples capped with quick-setting cement mortar were on average 10% higher than those capped with gypsum.

In the research the linear relationship between the compressive strength of mortars and their bulk density was found. Further experiments on other types of historical mortars should determine whether this relationship can be used to predict strength based on bulk density measurements.

In the study mortar samples in an air-dry state were tested. In heritage buildings, the moisture content of mortars in masonry joints can vary. The effect of moisture content in historical mortars on their strength characteristics is little understood and requires further research.

## Figures and Tables

**Table 1:** Characteristics of brick facilities and mortars.

Brick Facilities	Mortar	Building Erection Time	Elements from Which Mortar Samples Were Cut
B1	m1	60s of the 19th century	bridge arches
B2	m2	80s of the 19th century	external walls
	m3	50s of the 20th century	external walls
B3	m4	90s of the 19th century	external walls
	m5	second decade of the 20th century	internal walls
B4	m6	middle of the 20th century	external, internal walls

**Table 2:** Thickness of mortar samples.

Type of Mortar	n	*t*_*j*1_			*t*_*j*2_		
		Range of	Mean	CoV	Range of	Mean	CoV
		Values	Value		Values	Value	
	[-]	[mm]	[mm]	[-]	[mm]	[mm]	[-]
m1/g	12	12.6–19.8	16.7	0.14	18.2–24.5	21.1	0,10
m2/g	26	13.2–25.6	20.6	0.12	20.4–30.1	25.3	0.09
m3/g	18	14.7–25.8	20.7	0.18	21.1–32.6	25.5	0.13
m4/g m4/c	12 10	12.4–25.6 17.5–28.0	18.3 22.2	0.23 0.16	17.3–35.2 21.5–31.5	25.1 26.3	0.20 0.12
m5/g m5/c	12 9	16.1–26.0 19.9–26.1	21.4 22.2	0.18 0.08	21.0–32.8 24.0–32.8	27.0 26.0	0.14 0.11
m6/g m6/c	9 9	13.9–26.5 18.2–27.8	19.3 22.5	0.24 0.13	17.2–25.3 24.6–35.8	22.7 29.3	0.18 0.12

n—number of mortar samples; *t*_*j*1_—thickness of mortar sample equal to the thickness of masonry bed joint; *t*_*j*2_—thickness of sample in the central points (with capping layers); m…/g—samples with gypsum capping layers; m…/c—samples with cement mortar caping layers.

**Table 3:** Results of mortar strength tests.

Mortar Samples	*f_j_*				*d_max_*	Bulk Density (*)
	Range of Value	Mean Value	Stand.	CoV		
			Deviation			
	[MPa]	[MPa]	[MPa]	[-]	[mm]	[g/cm^3^]
m1/g	7.5–17.4	10.7	2.94	0.28	16	1.57
m2/g	1.4–3.4	2.4	0.51	0.22	12	1.42
m3/g	11.7–27.7	18.3	4.78	0.26	9	1.77
m4/g m4/c	2.8–7.2 3.9–9.1	5.1 6.0	1.35 1.69	0.26 0.28	7	1.55
m5/g m5/c	11.1–19.6 9.4–20.5	14.7 16.1	3.11 3.58	0.21 0.22	7	1.70
m6/g m6/c	3.1–6.8 2.9–7.8	4.4 4.9	1.36 1.60	0.31 0.32	11	1.59

(*) bulk density determined on mortar samples before capping with gypsum or cement mortar.

**Table 4:** Results of calculation mortar strength (f_jg_*) using regression functions.

Mortar Samples	*A* [MPa]	*m* [-]	f_jg_* (*t_j_*_1*min*_) [MPa]	f_jg_* (*t_j_*_1*min*_)/ f_jg_* (*t_j_*_1*mean*_)	f_jg_* (*t_j_*_1*mean*_) [MPa]	f_jg_* (*t_j_*_1*max*_) [MPa]	f_jg_* (*t_j_*_1*max*_)/ f_jg_* (*t_j_*_1*mean*_)	f_jg_* (*t_j_*_1*min*_)/ f_jg_* (*t_j_*_1*max*_)
m1/g	9.73	−0.31	11.2	1.09	10.3	9.8	0.95	1.14
m2/g	2.29	−0.31	2.6	1.13	2.3	2.1	0.91	1.24
m3/g	17.71	−0.027	17.9	1.02	17.8	17.6	0.99	1.02
m4/g	4.75	−0.35	5.6	1.14	4.9	4.4	0.90	1.27
m5/g	14.82	−0.59	16.8	1.17	14.4	12.7	0.88	1.32

**Table 5:** Evaluation of the mortar compressive strength (*f_m_*) based on the DPT test results.

Mortar	*t_j_*_1*mean*_ [mm]	*f_jg_* [MPa]	*f_m_* (6) [MPa]	*f_m_* (4) [MPa]	*f_m_* (7) [MPa]
m1/g	16.7	10.7	9.8	9.0	7.8
m2/g	20.6	2.4	2.3	2.3	1.8
m3/g	20.7	18.3	17.9	17.5	12.9
m4/g	21.4	5.1	4.8	4.8	3.9
m5/g	19.3	14.7	14.5	14.6	11.6

## Data Availability

The original contributions presented in this study are included in the article. Further inquiries can be directed to the corresponding author.
